# Gravitation field algorithm and its application in gene cluster

**DOI:** 10.1186/1748-7188-5-32

**Published:** 2010-09-20

**Authors:** Ming Zheng, Gui-xia Liu, Chun-guang Zhou, Yan-chun Liang, Yan Wang

**Affiliations:** 1 College of Computer Science and Technology, Jilin University, Changchun, 130012, China

## Abstract

**Background:**

Searching optima is one of the most challenging tasks in clustering genes from available experimental data or given functions. SA, GA, PSO and other similar efficient global optimization methods are used by biotechnologists. All these algorithms are based on the imitation of natural phenomena.

**Results:**

This paper proposes a novel searching optimization algorithm called Gravitation Field Algorithm (GFA) which is derived from the famous astronomy theory Solar Nebular Disk Model (SNDM) of planetary formation. GFA simulates the Gravitation field and outperforms GA and SA in some multimodal functions optimization problem. And GFA also can be used in the forms of unimodal functions. GFA clusters the dataset well from the Gene Expression Omnibus.

**Conclusions:**

The mathematical proof demonstrates that GFA could be convergent in the global optimum by probability 1 in three conditions for one independent variable mass functions. In addition to these results, the fundamental optimization concept in this paper is used to analyze how SA and GA affect the global search and the inherent defects in SA and GA. Some results and source code (in Matlab) are publicly available at http://ccst.jlu.edu.cn/CSBG/GFA.

## Background

Two of the most challenging tasks of optimization algorithms are to search the global optimum and to find all local optima of the space of solutions in clustering genes from available experimental data [1], e.g. the gene expression profiles, or given functions. In view of recent technological developments for large-scale measurements of DNA expression level, these two problems can often be formulated and many methods have been proposed. In particular, the heuristic searches are more promising than other kinds of searching approaches. These approaches include GA (genetic algorithm) [2], SA (simulated annealing) [3], PSO (Particle Swarm Optimization) [4] etc. But some inherent drawbacks, especially the inability to the multi-modal functions optimization, can be found from the traditional heuristic search algorithms above. Each of these concepts allows for several modifications, which multiplies the number of possible models for data analysis we can change the algorithm themselves, to find all the valleys of given functions. But we still have a lot of parameters to consider, as known as the number of valleys and the valley distance etc, and the performances of the modifications are not good enough.

GA is a traditional searching-optimization algorithm, this algorithm can search global optimal solution with probability criterion [5], but it can't converge to the global optimal solution in the theory [6]. So GA always traps in local optima or genetic drift. Anyway, the run-time of this algorithm is acceptable for most cases of searching-optimization problems.

SA is a generic probabilistic meta-algorithm for global optimization problems, namely locating a good approximation to the global optimum of a given function in a large search space. If we search enough time with SA, it will converge in the global optimal solution with probability 1 [7]. But the biggest drawback of SA is that the running-time of SA is so long that the efficiency is not tolerant to us.

Recently, some other searching algorithms have been proposed and discussed, such as PSO etc. These algorithms can search solution like GA. But actually, most of them will not converge to the global optimal solution either.

In this paper, we propose a new heuristic search approach Gravitation Field Algorithm (GFA). It not only can handle unimodal functions optimization, which traditional heuristic searches can do the same, but also can deal with multimodal functions optimization, which traditional heuristic search algorithms can't do. The experiments of the benchmark functions demonstrate that. The idea of GFA is derived from the modern widely accepted theory of planetary formation-- the Solar Nebular Disk Model (SNDM) [8] in the astronomy.

The complex astronomy theory can be introduced simply as follows:

Several billions years ago, there wasn't any planet in the Solar System; just dusts rounded the whole world. Then the dusts assembled by their own gravitations. For a long time, the rocks had come out. It was the threshold time of the whole Solar System. From then, the rocks moved fast to assemble together, and the bigger rocks arrested the smaller rocks. And they became larger rocks. Finally, the planets came out, and the rocks around them were absorbed out.

GFA is derived from the point of view of the hypothesis theory described above. To start with, all the solutions, which are the dusts in the algorithm model, are initialized randomly, or based on the prior knowledge; what's more, we assign every dust (solution) a weight, we call it mass, whose values are based on the mass function generated from the space of the problem solution; finally, the GFA begins. The power of attraction, which belongs to a certain dust and exists between every two dusts, pulls other dusts, which have the same influence to other dusts. Hence, the dusts assemble together, and the planets come out in the end--they are the optima. If you want to find global optimal solution, the planets assemble again, and the biggest planets will come out. To give a penalty of that the highest mass dust rules the whole space of solution, we propose a distance which can reduce the effect of gravitation field.

## Methods

### Description of GFA

Before all the works start, we design a mass function on the basis of special problem. The mass function is similar to the fitness function in GA. Both of them are score functions which are the criterions for a special solution. We can make the mass function be proportional to, or inversely proportional to, the extreme values of the problem. It all depends.

#### Initialization

The algorithm begins with the initial step. We generate and select n dusts d_i _(i = 1, ..., n) in the mass function domain [x_begin_, x_end_] to build the initial solution set. The positions of the dusts can be random or based on the prior knowledge (i.e. there will be greater probability to exist peak values in some positions). Then we assign a mass value to every dust. The mass values, which are described above, are associated with their positions, and are calculated by the mass function. So when the dusts move as we described below, they have a certain probability to find a new position, in which there is a new mass value that is bigger than the mass value of the centre dust. This initialization approach was used because the extension of the space solution could be considered. And the density of the dusts is proportional to the probability of existence of extreme value.

#### Strategy of division

**Figure 1:**
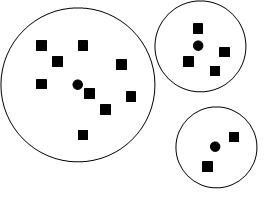
**Graph of power weight strategy in solution space**. The round black nodes are the centre dusts. The rectangle black nodes are surrounding dusts. The big white round charts with other nodes in them are the power distance domains of their internal centre dusts.

To decompose the solution space, we divide the space into pieces called groups. In one group, the special dust called centre dust corresponds to the max mass value in the group. The other dusts called surrounding dusts whose mass values are smaller than the mass value of the centre dust are in the power distance of the centre dust. The power distance is the space of the group in which the centre dust pulls other surrounding dusts toward it. De-signing an effective strategy of division is a challenging work in the GFA. When the number of in-dependent variables is greater than one, an appropriate strategy of division always needs a smart method. There are many criterions of division. For example, we can make a power weight strategy: the size of each group is decided by the mass value of the centre dust in the group, proportional or inversely proportional to the peak values. Fig. 1 was shown to explain this strategy.

Here, another simple average strategy of division in the form of two independent variables is given. The task of this strategy is to find the method of division in which the areas of all groups are all the same. First, we decide the number of groups m based on the mass function and the prior knowledge. Then we factorize m with two maximum factors, like Eq. (1):

(1) m=a×b 

The sizes of all groups are the same in this strategy. The power distance of each centre dust in the solution space is defined by Eq. (2):

(2) $$S=\frac{x}{a}\times \frac{y}{b}$$

Where x is the domain of x axis of the mass function; y is the domain of y axis of the mass function.

#### The rules of the motion of the dusts and strategy of absorption

The rules of the motion of dusts are the kernel part of GFA. This part of GFA decides which one is the peak value among all the dusts in one group. And this step of the algorithm also can generate new mass values which can have bigger mass values than the mass values of centre dust with certain probability.

Each group contains only one dust, called centre dust, that doesn't move within an epoch (but may move in other epochs). The rest dusts in this group move toward the centre dust within the epoch. Formally, centre (d_i_), where i = 1,..., n, is used to represent whether the dust d_i _is the centre dust or not: centre (d_i_) is set to true if d_i _is the centre dust; centre (d_i_) is set to false otherwise.

There are many kinds of strategies of motion. In this paper, the pace of the motion is set as Eq. (3):

(3) $$Pace_{i}=M\times dis_{i}$$

Eq. (3) is easy but efficient. dis_i _is the distance between the moving surrounding dust and the centre dust. M is a weight value for distance. 0.618/10 is chosen as M for this paper. Actually, many other weight values were tested in our experiment. But the speed and efficiency of GFA with the Golden Ration (0.618) are best. Maybe the value of it is not coincidence. But the reason, which is beyond our comprehension, will not mention in the paper. When the space of a group is small enough, the surrounding dusts will move with small pace calculated using Eq. (3); when the space of the group is big, surrounding dusts will move with big pace. Big pace will appear in two ways: One appears in the late algorithm. In this period, every dust in each group is quasi-optimal, big pace will not affect the efficiency of the GFA. So we make a big pace for fast convergence. The other one appears that we set a small number of dusts in the initial step. In this condition, maybe we want to make a fast convergence. And big pace could accelerate the convergence.

**Figure 2:**
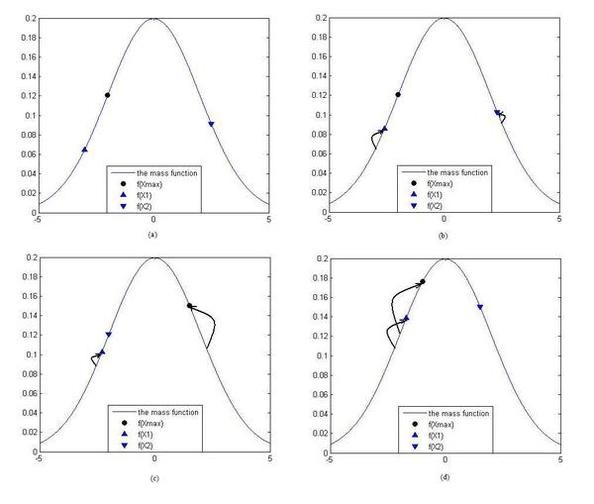
**The figures of the rules of the motion of the dusts**. The bell curve is the mass function. The round node is the mass value of the centre dust f (x_max_). The up triangle node is the mass value of surrounding dust x_1 _f(x_1_). The down triangle node is the mass value of surrounding dust x_2 _f(x_2_). Sub-figure (a) is a small group which has 3 dusts. Sub-figure (b) is the figure of motion of the surrounding dusts toward the centre dust. In the sub-figure (c), the mass value of the surrounding dust x_2 _is bigger than the mass value of the centre dust. And the centre dust becomes a surrounding dust which is coded x_2_; the surrounding dust x_2 _becomes the centre dust. In the sub-figure (d), the mass value of the surrounding dust x_2 _is bigger than the mass value of the centre dust. The surrounding dust x_2 _becomes the centre dust again.

When the surrounding dusts move toward the centre dust, the positions of the moving dusts will be changed. So the mass values of the surrounding dusts will also be changed. The diagrams of the motion of surrounding dusts in the form of one in-dependent variable are shown in Fig. 2. When the mass value of a moving surrounding dust becomes bigger than the centre dust and any other surrounding dusts, this surrounding dust will become a new centre dust as shown in Fig. 2 (b) and 2(c).

When all the distances between the surrounding dusts and the centre dust are small enough in one small group, such as smaller than a threshold, the surrounding dusts will be absorbed by the centre dust. Based on these rules of motion, we design the strategy of absorption as follows:

All the surrounding dusts are deleted, but the centre dust will not be. We set centre (d_i_) = false, it represents the small group for the next step. When the number of surrounding dusts is bigger than a threshold, we will absorb all the other surrounding dusts for saving running time.

After the absorption of the small groups is complete, the next epoch begins if the algorithm has not converged to the optimal solution. We will divide the space of the solution again, and compute for searching peak values using the survive dusts.

**Figure 3:**
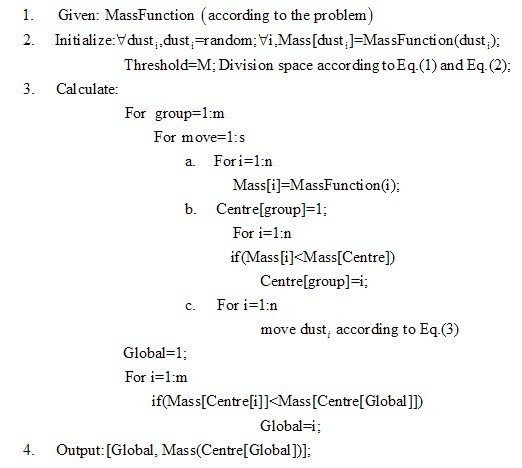
**The pseudo-code of a simple GFA**. In Fig 3, the number of dusts in one group is n, the number of groups is m, and the number of times of moving a dust is s.

The complete pseudo-code of a simple GFA is shown as Fig. 3.

### Mathematical framework

#### Mathematical proof

The most important advantage of GFA is the ability to deal with the multimodal objective functions (i.e. the mass functions). GFA can converge for one independent variable mass functions with probability 1. We give a theorem and its strict mathematical proof as follows.

We define the mass function as f(x), and the dusts in one group of variable space are x_1_, x_2_, ..., x_max_, ..., x_n_. The mass functions are subject to Eq. (4) as follows:

(4) $$f(x_{\max})\geq f(x_{m})\;$$

Where m = 1, 2, ..., n. After moving toward x_max _in the group, the dusts become x_1_', x2', ..., x_max_', ..., x_n_'.

First of all, we will give a description of groups and define two conceptions in the graph theory as follows:

**Definition 1**. *In Two Side (ITS): In the line segment of the x_m _and x_max _in the variable space, iff *∃ *x_m_', such that: f (x_m_')≥ f (x_max_'), then we call x_m _and x_max _are ITS*.

**Definition 2**. *In One Side (IOS): In the line segment of the x_m _and x_max _in the variable space, iff *∃ *x_m_', so that: f (x_m_')≥ f (x_max_'), then we call x_m _and x_max _are IOS*.

Now we give a theorem and prove it:

**Theorem 1**. GFA for one independent variable mass functions could converge in the global maximum with probability 1, when the three conditions as below are satisfied:

1) *The scale of group is small enough; such that the number of peaks is at most one*.

2) *The motion of the surrounding dusts is smoothing*.

3) *The number of dusts in one group is big enough*.

*Proof*. The formula form of condition 1) is Eq. (5):

(5) D(g)≤1

The number of dusts of group g is D(g). The max value of mass function in group g is f(x_max_), the real peak of the mass function in group g is f(x_peak_) (maybe the x_peak _isn't the dust in group g). Because the x_max _moves toward itself, it doesn't move. So Eq. (6) can be established.

(6) $$f(x_{\max})\equiv f(x_{\max}^{'})$$

**Figure 4:**
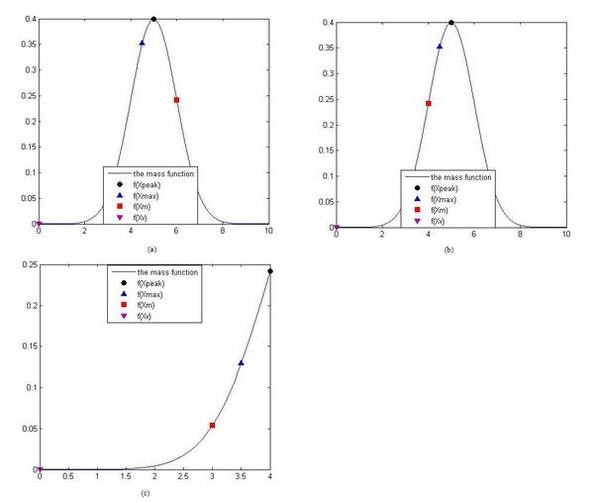
**The diagrams of the moving step**. f(Xpeak) is the peak value of the function in the group, the corresponding value in x axis is xpeak. f(Xmax) is the max value in the group, the corresponding value in x axis is xmax. f(Xm) is a mass value of a surrounding dust in the group, the corresponding value in x axis is x_m_. f(Xv) is the valley which is nearer than other valleys in the group, the corresponding value in x axis is x_v_. The sub-figure (a) is shown as the kind of the peak value must be found in the group. The sub-figure (b) and (c) are shown as the kind of the x_max _will be the pseudo peak value with probability p.

The convergence of one group can be proved as follows:

1) When x_max_ = x_peak_, the group is convergent obviously, f(x_max_) = f(x_peak_).

2) When the x_max_ ≠ x_peak_, then:

a) If exists x_m_, _max _and x_m _are ITS, the group will be convergent obviously according to condition 2), as Fig. 4 (a). The peak value calculated in the group by the GFA is the real peak value. We call this kind of condition is Real-Peak Condition (RPC).

b) If doesn't exist x_m_, such that the x_max _and x_m _are ITS, the group will not be convergent, the x_max _will be the pseudo peak value of the group, as Fig. 4 (b) and Fig. 4 (c). Pseudo peak value exists with probability p. we call this kind of condition is Pseudo-Peak Condition. (PPC)

The probability p can be calculated as follow:

We define the power distance of the center dust (see Description of GFA section) in the space of the solution in the group for one independent variable is l_total_. l_total _is shown as the domain of x axis in Fig. 4 (a), (b) or 4(c). And the distance between the x_max _and the nearest valley in the group for one independent variable is l_max_. l_max _is shown as the distance between x_max _and the x_v _in Fig. 4 (a), (b) or 4(c). f(x_v_) is the valley which is nearest x_max _in all valleys in the group.

When the PPC is on, there are n surrounding dusts in this group. So the n-1 dusts are in the space between x_max _and the nearest valley (i.e. the space of l_max_), and the locations of n-1 dusts are mutually independent. So when the strategy of initialization is used, i.e. all the dusts have equal possibility to exist anywhere, the probability of one dust is IOS with x_max _is l_max_/l_total _ So the formula of p is described as Eq. (7):

(7) $$p={\left(\frac{l_{\max}}{l_{total}}\right)}^{n-1}$$

Where P is the probability of all n-1 surrounding dusts are IOS with x_max_. From the Fig. 4, we know that l_max_ < l_total_, i.e. Eq. (8) can be established as follows:

(8) $$\frac{l_{\max}}{l_{total}}<1$$

So when condition 3) is satisfied, i.e. n → ∞, Eq. (7) can be:

(9) $$\underset{n\to \infty }{\lim}p=0$$

So when PPC is on, one group will converge in the real peak, like RPC is on. So every group will converge with probability 1.

When every group converges, the surrounding dusts are absorbed out. The real peak and pseudo peak will be the new dusts in the following step.

When X_max _becomes a dust in a new group of a new epoch, the global peak value could be X_max _in one group, obviously.

Hence in a certain domain of variable x, GFA will converge in the global maximum.

#### Peaks versus valleys

GFA maximizes the objective or mass function f(x). That is, they solve problems of the form:

(10) $$\underset{x}{peaks\;}\;f(x)$$

If you want to minimize f(x), like the experiments below, you can do so by maximizing -f(x), because the point at which the maximum of -f(x) occurs is the same as the point at which the minimum of f(x) occurs [9].

### Computation complexity of GFA

Computing the mass function value once needs processing time T_f_. Computing motion of every surrounding dust is T_m_. Computing the size relationship of every dust once needs processing time T_g_. We set the number of dusts in one group is n, the number of groups is m, and the number of times of moving a dust is s.

The computation complexity of processing mass function of every dust can be expressed by Eq. (11):

(11) $$Tf=T_{f}\times m\times n\times s$$

The computation complexity of Eq. (11) is o(mns).

The computation complexity of processing motion of every surrounding dust can be expressed by Eq. (12):

(12) $$Tm=T_{m}\times s\times m\times (n-1)\;$$

The computation complexity of Eq. (13) is o(mns).

(13) $$Tg=T_{g}\times m\times (n-1)\times s$$

The computation complexity of Eq. (14) is o(mns).

The computation complexity of GFA can be described as follows:

(14) $$T_{one}=Tf+Tm+Tg$$

Then we will find the smallest dust with all groups. The computation complexity of this process can be o(m). It's smaller than others. It can be ignored. So the computation complexity of GFA is:

(15) $$T=T_{f}\times m\times n\times s$$

If the complexity of mass function and the dimensionality of f(x) are big enough, we can say T = Tf. The number of dusts is m × n, so the computation complexity of GFA is the product of the number of dusts and the number of times of moving of surrounding dusts. That is, the computation complexity is o(mns).

## Results and Discussion

### Test Method

To test the efficiency of GFA, we assessed the performance of the GFA by employing a suite of different benchmark mathematical functions (Eqs. (16-20)) and by comparing the performance with GA and SA. For each of the five test functions and the each method, 500 minimization runs were performed and mean squared error, standard deviation and mean gauss error values were calculated.

#### Benchmark functions

In the following benchmark functions (Eqs. (16-20)), D donates the number of independent variables, and we defined D = 50. The benchmark functions were selected as following:

Sphere:

(16) $$f_{1}=\sum_{i=1}^{D}x_{i}^{2}$$

Rosenbrock:

(17) $$f_{2}=\sum_{i=1}^{D-1}\left(100{\left(x_{i}^{2}-x_{i+1}\right)}^{2}+(1-x_{i})\right)$$

Rastrigin:

(18) $$f_{3}=10D+\;\;\sum_{i=1}^{D}\left(x_{i}^{2}-10\cos(2\pi x_{i})\right)$$

Griewangk:

(19) $$f_{4}=\sum_{i=1}^{D}\frac{x_{i}^{2}}{4000}-\prod_{i=1}^{D}\cos(\frac{x_{i}}{\sqrt{i}})+1$$

Ackley:

(20) $$f_{5}=20+e-20e^{-0.2e^{\left(\sqrt{\frac{1}{D}\sum_{i=1}^{D}x_{i}^{2}}-e^{\frac{1}{D}\sum_{i=1}^{D}\cos(2\pi x_{i})}\right)}}$$

#### Error functions

Mean squared error (MSE) [10] for benchmark functions was calculated as Eq. (21):

(21) $$MSE=\frac{1}{n}\sum_{i=1}^{n}{\left(f(x_{i})-f_{opt}(x_{i})\right)}^{2}$$

Where n is the number of runs, f(x_i_) is the performance for run i and the f_opt_(x_i_) is the function value at the global minimum.

The standard deviation (STD) [11] was calculated as Eq. (22):

(22) $$STD=\sqrt{\frac{1}{n-1}\sum_{i=1}^{n}{\left(x_{i}-\bar{X}\right)}^{2}}\;$$

The gauss error (GE) function [12,13], which can be in the form of Eq. (23), is different from MSE:

(23) $$\text{GE}(x)=\frac{2}{\sqrt{\pi }}\int_{0}^{x}e^{-t^{2}}dt$$

GE is a partial differential equation which is twice the integral of the Gaussian distribution with 0 mean and variance of 1/2. And this function only can be used for a scalar. So we used it with average form called mean gauss error (MGE):

(24) $$\text{MGE}=\frac{1}{n}\sum_{i=1}^{n}GE(x_{i})$$

### Simulation Result

#### Searching global minimum

The implementation of GA and SA we used to compare with GFA is the Genetic Algorithm and Direct Search Toolbox™, which can be found from [14]. This toolbox was written with Matlab. Both the maximum number of epochs of GA and the initial temperature of SA were fixed to 1,000. For some functions, like Eq. (16), if the start point is fixed, the SA results of all the runs are all the same. So the initial start point of SA was randomly around the global minimum. A table that summarizes the parameter setting for each algorithm in each dataset was given as Table 1.

**Table 1:** Configuration of parameter setting for GFA, GA and SA.

Algorithm parameters	GFA	GA	SA
*Max. numbers of iterations*	1000	1000	1000
*Population size*	50	50	-
*Number of polulations*	200	200	-
*Initial temperature*	-	-	0~5.0
*Mutation rate*	0.02	0.02	-

To compare GFA with GA and SA, 500 minimization runs were performed on our suite of benchmark functions. And the domain of each dimension was defined as [-2, 2]. The comparison results were shown in Table 2.

**Table 2:** MSE, STD, and MGE of GA, GFA, and SA, Best performance (i.e., lowest error) for each function is highlighted in bold underline letters.

	Sphere	Rosenbrock	Rastrigin	Griewank	Ackley
**GA**					
MSE	7.2747	**0.0054**	7054.2	6.7709e-007	**14.6001**
STD	0.7409	**0.0556**	16.2621	6.8194e-004	**0.1891**
MGE	0.9927	0.0546	1	**5.2149e-004**	1
**GFA**					
MSE	**0.3347**	0.0156	**152.0279**	**5.3590e-007**	16.9460
STD	**0.2940**	0.0156	**6.5840**	**5.0375e-004**	16.9460
MGE	**0.4839**	0.1181	**0.9756**	6.0026e-004	1
**SA**					
MSE	1619.2	0.0069	745.7810	0.0030	26.9123
STD	7.1102	0.0827	12.5530	0.0385	26.9123
MGE	0.9967	**0.0061**	0.9952	0.0439	1

From Table 2, we found that sphere's, Rastrigin's and Griewank's functions could be optimized better by GFA than GA and SA, especially for the sphere's and Rastrigin's function.

Mean numbers of epochs were not same. It was an important criterion of the algorithm efficiency. The mean error served as success criterions along with a maximal number of epochs. If the threshold was not reached by an optimization method within 1,000 epochs, the run was judged as failure. No matter whether it was reached or not, the number of epochs that were needed to reach the threshold was rounded off and recorded in Table 3.

**Table 3:** Mean numbers of epochs until the minimization threshold was reached and mean number of failures.

	Sphere	Rosenbrock	Rastrigin	Griewank	Ackley
**GA**					
mean number of epochs	51	57	51	51	**92**
number of failures	0	0	0	0	0
**GFA**					
mean number of epochs	**31**	**46**	**30**	**49**	97
number of failures	0	0	0	**0**	0
**SA**					
mean number of epochs	816	46024	6349	21634	1001
number of failures	108	500	314	126	234

Best performance (i.e., lowest number) for each function is highlighted in bold underline letters.

GFA was able to outperform GA and SA in four of five benchmark functions in terms of epochs needed and least failures. Only in one of the five functions, GFA did not outperform in both speed and robustness. The minimization of the Ackley's function took slightly more epochs with the GFA (97) than with the GA (92). From the Table 3, we know that GA could do better for optimizing the complex function, such as Ackley's function. But for some simple function, such as sphere's function, GFA could do better.

For more accurate computation, we defined the initial number of dusts 10,000, the max power distance of center dust 5, and the epochs 1,000, which was the same as used in GA and SA (for more information of detail parameters, please see description of GFA).

Overall, The GFA achieved a large decrease on the MSE compared to the GA for some functions (De Jong's Sphere: 21.735-fold; Rastrigin: 46.4007-fold). Relatively simple functions could be optimized better than GA and SA through the phenomenon of the experiment. The efficiency of SA was poorly in the experiment. And the run-time of GFA and GA were in the same magnitude, but the run-time of SA is much longer than the two others. We can see the total running-time from Table 4.

**Table 4:** total running-time of GFA, GA and SA with 500 runs.

	Sphere	Rosenbrock	Rastrigin	Griewank	Ackley
**GA**	**187.13**	**63.29**	157.33	**63.98**	101.30
**GFA**	201.37	67.90	**113.46**	69.42	**84.59**
**SA**	14211.08	11463.29	52914.75	15536.02	6406..68

Smallest running-time for each function is highlighted in bold underline letters. 2.66 GHz 2 cores Intel CPU and 2 G memories are in the computer used to calculate Eqs. (16-20) with GA, GFA and SA. The unit of the running-time is second.

#### Searching multi-minima

Although minimum of certain given function could be optimized to resolve the problem for most situations, minimum is not always what we want only. Sometimes a part of minima is also needed. Bayesian network inferring, for example, is a stochastic approach. So the minimum of Bayesian criterion function is not always fit the realistic network best. It is important to find other minima for the problems as cases described above. A number of lowest minima can be retained in the search.

To run multimodal optimization algorithm, we must track more information than the global optimization. Searching exactly a certain number of minima or even all of the minima in the domain is a challenging work, which is also meaningful for some problems, e.g. gene cluster. In the experiment, top 5 minima were searched with GFA. It's beyond GA's and SA's ability.

Because sphere's function is a unimodal function, we could not use it for search multi-minima. We use the rest of the four benchmark function. To complete the experiment, 500 optimization runs were performed on each benchmark functions. The settings were shown as Table 5.

**Table 5:** GFA settings configurations of multi-minima optimization

	Rosenbrock	Rastrigin	Griewank	Ackley
Domain of each variable	[-2,2]	[-1.5,1.5]	[-1,1]	[-2,2]
Max numbers of iterations	1000	1000	1500	2000
number of dusts	10000	10000	15000	20000
Number of groups	200	200	300	400

The direct result values of the multi-valleys searching with GFA are shown in Table 6. From Table 6, it can be seen that for all multimodal functions the searching results can reach high precision. It means that the stability of GFA for multi-minima optimization is very good.

**Table 6:** Top 5 mean minima value of 500 runs for each benchmark function

Minima Index	Rosenbrock	Rastrigin	Griewank	Ackley
1	1.0029	1.0470	0.0112	3.9736
2	0.1018	1.2767	0.0083	3.524
3	0.0030	0.0706	0.0006	0.0032
4	0.1038	1.0313	0.0079	3.623
5	1.0033	1.0200	0.0102	3.6535

### Application to gene cluster

GFA could be applied in many science research areas, especially bioinformatics. We used it for gene cluster. The clustering algorithm we used was K-means. The dataset used in this paper was from the experiment of Spellman [15]. And the data which was MIAME compliant [16] could be downloaded from GEO with accession number GDS38. The number of cases was 7,680, of which the 17 missing values were excluded. We computed SMBS correlation coefficient [17] and excluded missing values with Matlab. We excluded cases when the respondent was dropped only on analyses involving variables that had missing values. The data with 7663 cases and 16 samples was divided into 20 parts, as known as clusters. So we distributed the cases into 20 groups. And our mission was that make sure the mean distance was smallest or the number of runs exceeded 1,000. To compare with GFA, 3 methods, GA, SA and Cluster 3.0 [18], were used to test the efficiency of the novel algorithm. K-means was used in Cluster 3.0 with correlation (centered) coefficient. For there four clustering methods, 500 runs were performed. And we got the mean value.

To visualize the result of the gene cluster, we used the free software TreeView [19] from Eisen's Lab. A part results computed by the novel algorithm and other cluster methods of gene cluster were showed in a picture which is Additional file 1. This picture shows the C8.txt and result.txt with Group = 7, which should be opened with excel software.

But there is no single best criterion for obtaining a partition because no precise and workable definition of cluster exists. Clusters can be of any arbitrary shapes and sizes in a multidimensional pattern space [20]. It is impossible to objectively evaluate how good a specific clustering method is without referring to what the clustering will be used for [21]. So we evaluate the result with F test with only mathematical aspect. And P values of all the 16 samples are less than 0.01. The total results of the gene cluster with GFA could be downloaded from our website.

In the Bioinformatics, we could check the biologically meaning. Correlations between cis elements and expression profiles could be established within the novel algorithm cluster result. The cis elements we used were from [22]. It's generated by Li-Hsieh Lin etc [23]. We compare GFA with GA, SA and Cluster 3.0 in C8 and summarized it in Table 7.

**Table 7:** cis-regulatory elements correspond each method

	Fkh1	Fkh2	Ndd1	Mcm1	Ace2	Swi5	Mbp1	Swi4	Swi6
GFA	1	3	4	2	0	5	2	3	2
Cluster 3.0	1	1	0	0	1	0	0	3	2
GA	3	4	3	2	1	4	4	2	1
SA	0	3	2	1	1	2	3	4	1

From Table 7, the efficiency of GFA could be seen. The genes with the same cis-regulatory elements could be clustered better by GFA than Cluster 3.0 and SA. Only in the case of the Ace2 cis element, the GFA with optimized parameters could not outperform the Cluster 3.0. Some efficiency is very obvious, especially Ndd1 and Swi5. But it seams that the efficiency of GA and GFA are the same. The results of this cluster experiment indicate that GFA method does work in gene cluster with finding cis-regulatory element in the same cluster. Other clustering results have the same properties.

### Software

The developed algorithm software of single-machine for GFA was implemented with matlab R2008a in the software package GFA, which is a short code file, freely available from our website: http://ccst.jlu.edu.cn/CSBG/GFA. You can implement the multi-machines parallel algorithm for GFA based on the detail in the conclusion section below.

## Conclusions

In this paper we propose a generalization searching-optimization algorithm called GFA. This algorithm derives from the SNDM theory, and the efficiency of the algorithm is shown as above. We can summarize them into three parts:

In the form of one independent variable, the GFA will converge with probability 1. It gives us a balance level between time and efficiency. If you want to find exactly extreme value of the mass function, you can disperse more dusts to avoid the condition of the p (see mathematical proof). If you want to find the extreme value fast, you can define a small number of initial dusts.

GFA can find all needed the peaks of the solution. No matter the number of initial dusts is big or small.

The running-time of GFA is very short. The reasons are the strategy of the division and the rules of motion described as above. The space of solution is cut into small groups. It's the decomposition of any complex problem. Even more, it will support us a feasibility of parallel computing. In this view, this algorithm is similar to the parallel genetic algorithm [24]. But this mechanism of GFA can be faster than GA's. We could use a large number of idle computers to calculate a complex problem, and the running-time is inversely proportional to the number of the computers.

## Competing interests

The authors declare that they have no competing interests.

## Authors' contributions

MZ designed the algorithms, carried out the experiments and drafted the manuscript. CGZ and GXL conceived and coordinated the research, participated in the design of the experiments and carried out parts of the experiments. YCL and helped to draft the manuscript. All authors read and approved the final manuscript.

## Supplementary Material

Additional file 1 **A picture in which is a graph of a part of clusters with the real gene expression dataset GDS38**. In the picture, there are four graphs which were computed by GFA(a), Cluster 3.0(b), GA(c) and SA(d) with the K-means clustering algorithm all. They were corresponded the same cluster in the 20 ones. Red represents positive, green represents negative, black represents zero and grey represents missing values. Click here for file
