# Supplementary figures and images for: Gravitation field algorithm and its application in gene cluster
Source: Algorithms Mol Biol. 2010 Sep 20;5:32. doi: 10.1186/1748-7188-5-32 (PMC2949600; doi:10.1186/1748-7188-5-32)

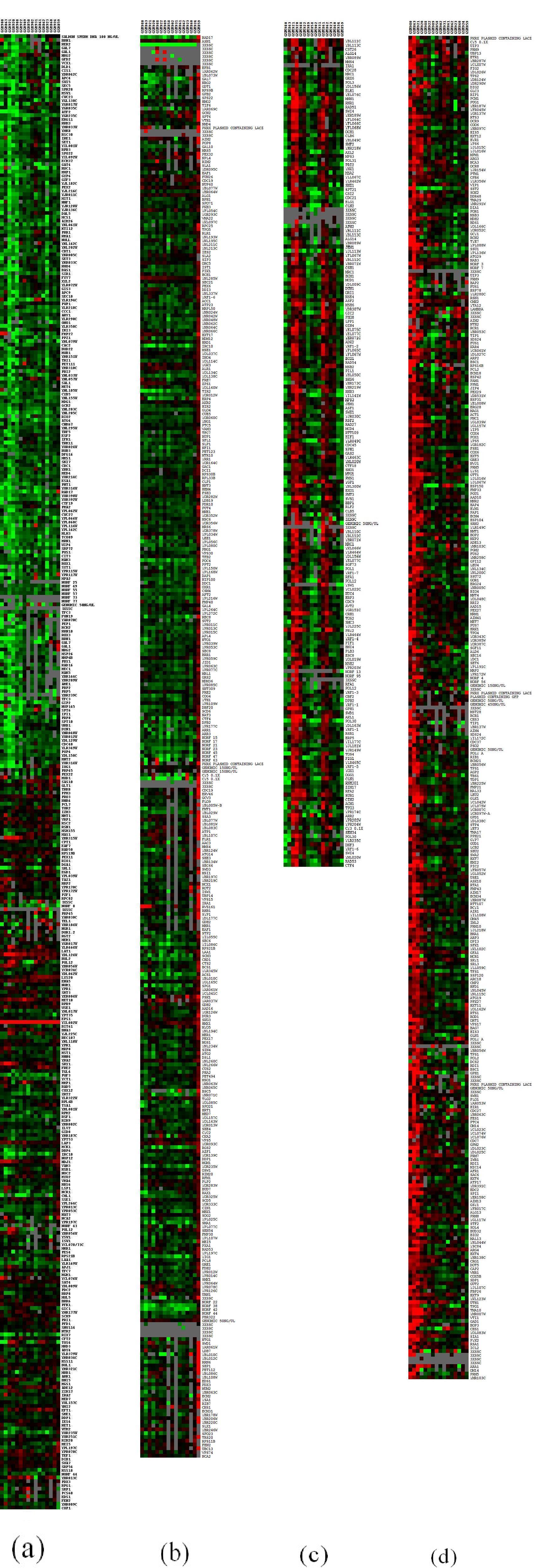

Supplement: Additional file 1 — A picture in which is a graph of a part of clusters with the real gene expression dataset GDS38. In the picture, there are four graphs which were computed by GFA(a), Cluster 3.0(b), GA(c) and SA(d) with the K-means clustering algorithm all. They were corresponded the same cluster in the 20 ones. Red represents positive, green represents negative, black represents zero and grey represents missing values. [file 1748-7188-5-32-S1.JPEG]
